# Targeting BTK in B Cell Malignancies: From Mode of Action to Resistance Mechanisms

**DOI:** 10.3390/ijms25063234

**Published:** 2024-03-12

**Authors:** Samir Mouhssine, Nawar Maher, Bassam Francis Matti, Alaa Fadhil Alwan, Gianluca Gaidano

**Affiliations:** 1 Division of Hematology, Department of Translational Medicine, Università del Piemonte Orientale and Azienda Ospedaliero-Universitaria Maggiore della Carità, 28100 Novara, Italy; samir.mouhssine@uniupo.it (S.M.); 20024416@studenti.uniupo.it (N.M.); 2 Department of Hematology and Bone Marrow Transplant, Hematology and Bone Marrow Transplant Center, Medical City, Baghdad 00964, Iraq; bassam_francis@yahoo.com (B.F.M.); ala_sh73@uomustansiriyah.edu.iq (A.F.A.); 3 Department of Clinical Hematology, The National Center of Hematology, Mustansiriyah University, Baghdad 10015, Iraq

**Keywords:** chronic lymphocytic leukemia, mantel cell lymphoma, lymphoplasmacytic lymphoma, diffuse large B cell lymphoma, Bruton tyrosine kinase, BTK degraders

## Abstract

The B cell receptor (BCR) signaling pathway plays a crucial role in B cell development and contributes to the pathogenesis of B cell neoplasms. In B cell malignancies, the BCR is constitutively active through both ligand-dependent and ligand-independent mechanisms, resulting in continuous Bruton tyrosine kinase (BTK) signaling activation, which provides a survival and proliferation advantage to the neoplastic clone. Among B cell malignancies, those in which the most significant results were obtained by treatment with BTK inhibitors (BTKi) include chronic lymphocytic leukemia, mantle cell lymphoma, lymphoplasmacytic lymphoma, and diffuse large B cell lymphoma. Covalent BTKi (namely ibrutinib, acalabrutinib, and zanubrutinib) functions by irreversibly blocking BTK through covalent binding to the cysteine residue 481 (Cys-481) in the ATP-binding domain. Despite the high efficacy and safety of BTKi treatment, a significant fraction of patients affected by B cell malignancies who are treated with these drugs experience disease relapse. Several mechanisms of resistance to covalent BTKi, including Cys-481 mutations of BTK, have been investigated in B cell malignancies. Non-covalent BTKi, such as pirtobrutinib, have been developed and proven effective in patients carrying both Cys-481-mutated and unmutated BTK. Moreover, targeting BTK with proteolysis-targeting chimeras (PROTACs) represents a promising strategy to overcome resistance to BTKi in B cell neoplasms.

## 1. Introduction

B cell malignancies are a heterogeneous group of hematologic neoplasms characterized by the abnormal proliferation of malignant B lymphocytes and include the majority of lymphomas, several types of leukemia, and multiple myeloma (MM) [1,2]. Although chemoimmunotherapy (CIT) has represented the standard of care in B cell malignancies, in recent years, targeting Bruton tyrosine kinase (BTK) with innovative drugs, namely BTK inhibitors (BTKi), has revolutionized the therapeutic landscape for these diseases [3]. Currently, BTKi can be distinguished into covalent and non-covalent BTKi, according to the precise mode of action [4,5].

The B cell receptor (BCR) signaling pathway plays a crucial role in B cell development and contributes to the pathogenesis of B cell neoplasms [6]. The activation of BCR signaling occurs when an antigen binds to the surface immunoglobulin (sIg), leading to the coupling and autophosphorylation of immunoreceptor tyrosine-based activation motifs (ITAM) on the cytoplasmic tails of CD79A (Igα) and CD79B (Igβ) by the protein kinase LYN, which belongs to the Src family [7]. Subsequently, ITAM phosphorylation creates docking sites for the tyrosine kinase SYK, which activates the B cell linker scaffold protein BLNK [8]. As a consequence, BTK is activated through phosphorylation at its aminoacidic Y551 residue by either LYN or SYK [8]. Upon activation, BTK initiates several downstream signaling pathways, such as the phosphoinositide 3-kinase (PI3K)-AKT pathway and phospholipase Cγ2 (PLCγ2) pathway, leading to the activation and nuclear migration of various transcription factors, including mTOR, NF-κB, ERK1/2, and NFAT [8,9]. These pathways are involved in switching on cellular programs relevant for the survival, differentiation, and proliferation of B cells [10]. Additionally, BTK can be triggered by other receptors, such as growth factors, cytokine receptors, and G-protein-coupled receptors (GPCRs), including chemokine receptors and integrins [11].

In B cell malignancies, the BCR is constitutively active through both ligand-dependent and ligand-independent mechanisms, resulting in continuous BTK signaling activation [12]. This persistent activation provides a survival and proliferation advantage to the neoplastic clone of B cell neoplasms [13]. Among B cell malignancies, those in which the most significant results were obtained by BTK targeting are chronic lymphocytic leukemia/small lymphoma (CLL/SLL), mantle cell lymphoma (MCL), lymphoplasmacytic lymphoma (LPL), and diffuse large B cell lymphoma (DLBCL) [3,14,15,16].

CLL is a hematologic neoplasm marked by the clonal proliferation of mature B cells [1,2]. The clinical diagnosis of CLL requires a lymphocyte blood count ≥ 5 × 10^9^/L, along with a specific immunophenotypic profile that includes the clonal expression of CD19, CD5, CD20, CD23, and sIg [2]. CLL is the most common leukemia in adults [17]. The disease has a low risk of progression and relatively low lethality, with a 5-year relative survival rate approaching 90% [18]. Importantly, CLL predominantly affects elderly individuals, with a median age at diagnosis of 72 years in high-income countries (HICs) [17,18]. Despite its indolent clinical course and extended overall survival (OS), CLL is not completely curable, and patients refractory to currently available therapies face the risk of disease progression and, eventually, disease-related mortality [13]. This emphasizes the need to address treatment refractoriness in CLL and to develop novel therapeutic approaches. The current standard of care for CLL includes a variety of treatment options with pathway inhibitors, targeting either the BTK or the BCL2 pathways, to manage the disease and improve patient outcomes [14]. Ongoing research efforts aim to uncover new treatment modalities, understand the underlying biology of CLL, and explore innovative therapeutic strategies to enhance the management of this hematologic malignancy.

MCL is a distinct and relatively aggressive subtype of B cell non-Hodgkin lymphoma (NHL) that accounts for approximately 5% of all NHL cases [1,2,19,20]. MCL typically affects individuals in their mid-60s and is characterized by the t(11;14)(q13;q32) translocation, leading to the overexpression of cyclin D1, which is a major regulator of cell cycle progression from the G_1_ to S phase [21,22]. This genetic alteration results in uncontrolled cell cycle progression, contributing to the aggressive nature of MCL [21]. The clinical presentation of MCL often involves lymph node enlargement, splenomegaly, and bone marrow involvement [1]. MCL can exhibit systemic dissemination, with extranodal involvement commonly observed in the gastrointestinal tract [23]. Treatment approaches for MCL are tailored based on various factors, including the patient’s age, performance status and comorbidities, and disease characteristics [24]. Intensive chemotherapy regimens, immunotherapy, and autologous stem cell transplantation (ASCT) are commonly employed in young and fit transplant-eligible patients. Notably, the introduction of novel agents has transformed the treatment landscape for MCL; in particular, BTKi have demonstrated significant efficacy, providing a targeted therapy for MCL patients [25,26,27,28].

LPL, also known as Waldenström’s macroglobulinemia (WM), is a B cell neoplasm characterized by the clonal expansion of small lymphocytes, plasma cells, and plasmacytoid lymphocytes in the lymphoid tissues, particularly in the bone marrow [1,2]. This indolent NHL is characterized by the presence of a monoclonal immunoglobulin M (IgM) in the serum, which is a key diagnostic feature [29]. More than 90% of LPL cases harbor the L625P activating mutation of the *MYD88* gene [30]. The pivotal involvement of *MYD88* in LPL pathogenesis is evident in its enhancement of Toll-like receptor signaling, ultimately activating transcription factors belonging to the NF-κB family and associated with the growth and survival of both normal and neoplastic B cells [30,31]. LPL commonly involves the bone marrow, leading to cytopenias and bone marrow failure, and might manifest systemically, affecting various organs such as the spleen and liver [29]. Common clinical manifestations of LPL include B symptoms, bruising, fatigue, malaise, and clinical manifestations related to blood hyperviscosity [32]. Management strategies for LPL depend on the clinical presentation and disease burden [33]. Asymptomatic or minimally symptomatic cases may be subjected to watchful waiting with periodic assessments. Symptomatic cases or those with progressive disease may require systemic therapies, including chemotherapeutic agents, immunomodulatory drugs, or targeted therapies like BTKi [29,34,35].

DLBCL is an aggressive, highly heterogeneous non-Hodgkin lymphoma (NHL) that represents the most common subtype of lymphoma [2]. Most patients present with generalized lymphadenopathy. Also, extranodal involvement is found in about 30% of patients, most commonly involving the gastrointestinal tract, bone, testes, spleen, central nervous system, and other sites [36]. DLBCL is a potentially curable disease with an OS of 60–70%, with the currently used first-line CIT consisting of R-CHOP [37]. Nevertheless, 30–40% of the patients are either refractory to the first-line treatment or experience relapse (R/R).

DLBCL, based on gene expression profiles, is divided into activated B cell-like (ABC-DLBCL) and germinal center B cell (GCB-DLBCL) types, with each group representing a differentiation state [38]. The ABC subtype is characterized by persistent activation of the NF-κB signaling cascade following stimulation of the BCR pathway, thus providing a rationale for experimental BTKi therapy targeting. In contrast, GCB-DLBCL shows an increased dependence on the PI3K and BCL2 signaling pathways [39,40]. In the era of precision medicine, extensive efforts have been dedicated to subclassifying DLBCL based on genetic and biological characteristics, which holds promise for targeted therapeutic interventions. Among the notable molecular subgroups within this framework are the MYD88-driven subtype (MCD), characterized by the enrichment of gain-of-function mutations in *MYD88* L265P and/or *CD79B*, and the N1 subtype distinguished by *NOTCH1* mutations [41,42,43].

The extensive use of BTKi in multiple B cell malignancies over the years has also led to clinical refractoriness in a fraction of cases, prompting the search for the molecular mechanisms underlying clinical refractoriness [13,44]. On these grounds, the aim of this review is to summarize the mode of action of BTKi and the mechanisms of resistance to BTK targeting in B cell malignancies, with a particular focus on CLL, MCL, LPL, and DLBCL.

## 2. Covalent BTK Inhibitors

The first-in-class covalent BTKi is ibrutinib, a highly potent, first-generation BTKi approved by the Food and Drug Administration (FDA) in 2013 for the treatment of MCL [45]. Covalent BTKi function by irreversibly blocking BTK through covalent binding to the cysteine residue 481 (Cys-481) in the ATP-binding domain [13,46]. This action results in the occupation of the ATP-binding site, preventing the phosphorylation of downstream targets like Akt and PLCγ2 [13]. Consequently, BTK signaling is hindered, leading to the inhibition of the BCR pathway observed both in vitro and in vivo [47]. Apart from this intended on-target effect, ibrutinib also deactivates several off-targets, including EGFR, ErbB2, ITK, and TEC [48,49]. While contributing to the anti-tumor effect, these off-target effects are associated with adverse events, such as atrial fibrillation, bleeding, and the impairment of macrophage phagocytosis [48,49,50].

More recently, second-generation BTKi, namely acalabrutinib and zanubrutinib, were developed and approved for the treatment of B cell malignancies [51,52,53]. Acalabrutinib and zanubrutinib are irreversible and highly effective BTKi, exhibiting greater selectivity than ibrutinib for the Cys-481 residue in the binding site. This results in the reduced off-target inhibition of other kinases in the TEC family, including EGFR and ITK, leading to fewer adverse events [54,55]. Compared to ibrutinib-based therapy, treatment with second-generation BTKi is burdened by a lower incidence of cardiovascular adverse events, such as atrial fibrillation/flutter and bleeding [54,55,56]. In particular, a recent meta-analysis that compared standard treatment versus second-generation BTKi therapy in B cell malignancies reported no difference in death rate due to cardiovascular adverse events [57].

### 2.1. Covalent BTK Inhibitors in CLL 

The introduction of ibrutinib for CLL treatment marked the beginning of the era of kinase-targeted drugs for this disease [58]. In 2014, approval for the use of ibrutinib for the treatment of CLL was granted based on the outcomes of the pivotal Phase 1b/2 trial PCYC 1102, which highlighted its effectiveness in individuals with untreated and relapsed/refractory (R/R) CLL/SLL [58,59]. With a median follow-up of 26 months, the estimated 7-year progression-free survival (PFS) reached 83% for treatment-naïve patients and 34% for R/R patients, with an overall response rate (ORR) of 89% [59]. In the context of R/R disease, ibrutinib demonstrated superiority (in terms of PFS and OS) over ofatumumab in the RESONATE trial [60]. Additionally, as a frontline therapy, ibrutinib outperformed chlorambucil in terms of PFS and OS in the RESONATE-2 trial [61]. Further validation of ibrutinib efficacy was provided by the Alliance trial (A041202), which assessed the efficacy of ibrutinib (alone and in combination with rituximab) against chemoimmunotherapy (bendamustine–rituximab, BR) [62]. Similarly, the E1912 trial compared the ibrutinib–rituximab regimen with chemoimmunotherapy (fludarabine, cyclophosphamide, and rituximab, FCR) in young and fit patients [63]. Both studies indicated prolonged PFS for patients treated with ibrutinib, with the ibrutinib-treated patients in E1912 experiencing extended OS [62,63].

Acalabrutinib, with or without the anti-CD20 monoclonal antibody (mAb) obinutuzumab, demonstrated superior performance compared to CIT regimens in treatment-naïve and R/R CLL [64,65]. Notably, this efficacy superiority was maintained in IGHV unmutated patients and TP53-disrupted patients, representing disease categories associated with biomarkers of poor prognosis [64]. The SEQUOIA trial demonstrated the superiority of zanubrutinib over BR, with significantly improved PFS in non-del(17p) patients. The ALPINE trial, a head-to-head comparison of zanubrutinib and ibrutinib, revealed a higher overall response rate (ORR) (78.3% vs. 62.5%) and 12-month OS rates (97% vs. 92.7%) [55,66].

### 2.2. Covalent BTK Inhibitors in MCL

In recent years, BTKi, notably ibrutinib, have emerged as promising treatment options for MCL [67]. A single-arm phase II trial explored the efficacy of ibrutinib and rituximab (IR) induction followed by R-HyperCVAD/MA (rituximab, cyclophosphamide, vincristine, doxorubicin, dexamethasone/methotrexate, cytarabine) in 131 patients, reporting a 98% ORR with 87% complete response (CR) after a median follow-up of 42 months [68]. The phase III TRIANGLE trial evaluated ibrutinib in induction and maintenance therapy in 870 patients randomly assigned to three arms [69]: (i) arm A, in which patients received an induction regimen comprising three cycles of R-CHOP/R-DHAP followed by ASCT; (ii) arm A + I, consisting in induction therapy with the incorporation of ibrutinib into three cycles of R-CHOP/R-DHAP, followed by a two-year maintenance phase with ibrutinib; (iii) arm I, which involved a combination approach, utilizing ibrutinib alongside the induction therapy of R-CHOP/R-DHAP, followed by a two-year maintenance period with ibrutinib, omitting ASCT [69]. Arm A + I showed an improved failure-free survival (FFS) reaching 88%, compared to 72% in arm A, after a median follow-up of 31 months. Three-year OS rates were 86%, 91%, and 92% for arms A, A + I, and I, respectively. Notably, ibrutinib-containing treatments without ASCT demonstrated a safety profile comparable to standard regimens [69].

Recently, the covalent BTKi zanubrutinib was approved for the treatment of MCL based on the promising results obtained in phase II trials, and phase III trials with this drug are now ongoing [28,70]. The ongoing phase III study NCT04002297 is comparing zanubrutinib plus rituximab followed by zanubrutinib monotherapy versus BR, followed by observation in transplant-ineligible, previously untreated MCL patients [71]. Moreover, in a phase II trial involving R/R MCL patients, promising results were obtained with acalabrutinib monotherapy, displaying an ORR and CR rate of 81.5% and 47.6%, respectively [27]. Over a median follow-up duration of 38.1 months, the median PFS was 22.0 months, and the estimated median OS was 59.2 months. Remarkably, patients with blastoid/pleomorphic histology, which represents a risk factor for a more aggressive and refractory disease, achieved an ORR of 80.8%, similar to that of the general population [27].

### 2.3. Covalent BTK Inhibitors in LPL 

The approval of ibrutinib has changed the treatment landscape for LPL, ushering in a chemotherapy-free approach [72]. Several clinical trials highlight ibrutinib’s efficacy as a single agent in LPL. In a phase II study dedicated to previously treated patients, ibrutinib achieved a 91% ORR and 73% major response rate (MRR), with rapid minor and major responses in 1 and 2 months, respectively [73]. Additionally, the INNOVATE study, a phase II trial combining ibrutinib with rituximab, demonstrated superior ORR and major response rates compared to rituximab alone [74].

Although the advent of ibrutinib marked a paradigm shift in LPL treatment, resulting in notable improvements in patients’ quality of life, adverse events (AEs), including atrial fibrillation, have raised concerns [75,76]. In addition to the lower cardiovascular toxicity displayed by zanubrutinib, its pharmacokinetics are unaffected by renal dysfunction and hepatic impairment, ensuring flexibility in cases with severe hepato-renal alterations [77]. Monotherapy studies in treatment-naïve and R/R patients underscored its tolerability and deep, durable responses across molecular subtypes, even in *MYD88* wild-type (*MYD88*^wt^) cases that otherwise responded poorly to ibrutinib monotherapy [73,74,77,78]. The phase III ASPEN study compared zanubrutinib and ibrutinib in LPL, emphasizing zanubrutinib’s superior safety, with lower atrial fibrillation and improved overall tolerability [78].

In a phase II trial that enrolled 106 LPL patients, comprising 14 treatment-naïve and 92 previously treated cases, acalabrutinib monotherapy obtained an ORR of 93% [79]. MRR was 79% for treatment-naïve and 78% for previously treated patients. Similarly to zanubrutinib, acalabrutinib achieved a higher ORR and MRR in *MYD88*^wt^ LPL patients compared to ibrutinib. Their favorable safety and efficacy profile position zanubrutinib and acalabrutinib as compelling, long-term single-agent options for the treatment of LPL, providing a significant therapeutic advancement in this challenging disease.

### 2.4. Covalent BTK Inhibitors in DLBCL

Patients diagnosed with ABC-DLBCL have significantly poorer survival outcomes when treated with standard R-CHOP therapy compared to those presenting with the GCB subtype [39]. Given the chronic activation of the BCR signaling pathway in ABC-DLBCL, clinical studies commenced with the stratification of DLBCL based on subtype determination. In a pivotal phase 1/2 trial (NCT00849654, NCT01325701), ibrutinib displayed notable efficacy, primarily in the ABC subtype, demonstrating an overall response rate (ORR) of 37% as opposed to 5% in individuals with the GCB subtype [80]. Based on these grounds, the phase III PHOENIX trial investigated the efficacy of ibrutinib compared to placebo in combination with first-line R-CHOP in patients with non-GCB subtype disease. However, adding ibrutinib to R-CHOP did not demonstrate an OS benefit in the study population as a whole [81]. A subgroup analysis revealed improvements in event-free survival (EFS), PFS, and OS that were limited to the <60-year-old cohort. The study showed that EFS and OS exhibited increases of 10.8% and 12.3%, respectively, in the ibrutinib arm compared to the control arm. The lack of benefit observed in older patients was partly attributed to increased treatment-related toxicity with the combination therapy, leading to a suboptimal dosing of CIT. 

A subsequent subgroup analysis focusing on younger patients (age ≤ 60) revealed that patients with ABC-DLBCL of the MCD and N1 subtypes experienced a notable 3-year EFS and OS rate of 100% when treated with ibrutinib plus R-CHOP, in contrast to 42.9% and 50%, respectively, in the R-CHOP-alone arm [82]. Although this study was not statistically designed to compare responses or survival outcomes among these subgroups, the results are hypothesis-generating and underscore the need for larger studies to identify genetic subgroups that may derive greater benefits from BTKi-containing therapy. According to a recent study, the high sensitivity of the MCD-DLBCL subtype to ibrutinib is attributed to a non-canonical form of chronic selective autophagy [83]. Specifically, this autophagic process targets ubiquitinated MYD88 L265P for degradation in a tank-binding kinase 1 (TBK1)-dependent manner within MCD-DLBCL. TBK1 is a crucial serine/threonine kinase involved in various physiological cellular processes, including selective autophagy and innate immunity regulation. However, MCD tumors undergo genetic and epigenetic alterations that attenuate this autophagic tumor-suppressive pathway. Conversely, BTKi promote the autophagic degradation of MYD88 L265P, thereby elucidating their clinical benefit in MCD-DLBCL and suggesting the evaluation of autophagy inhibitors as future therapeutic agents.

## 3. Mechanisms of Resistance to Covalent BTK Inhibitors

Despite the high efficacy and safety of BTKi treatment, a significant fraction of patients affected by B cell malignancies treated with these drugs experience disease relapse [13,44]. The mechanisms of resistance have been investigated in CLL, MCL, and LPL, while little is known about BTKi resistance in other B cell malignancies. However, different primary and acquired mechanisms have been described to cause resistance to both covalent and non-covalent BTKi.

**Figure 1:**
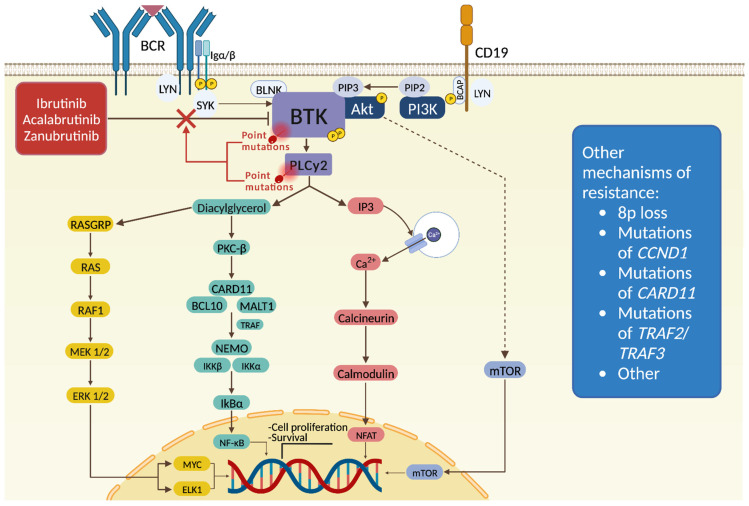
Acquired resistance mechanisms to covalent BTKi. Upon binding to antigens, the BCR triggers the formation of a signaling complex by phosphorylating immunoreceptor-based activation motif (ITAM) residues on CD79A (Igα) and CD79B (Igβ) cytoplasmic tails. This activation leads to SYK activation, subsequently activating BTK, PLCγ2, and PI3K. Downstream responses encompass culminates in the activation of NF-κB, ERK1/2, NFAT, and mTOR transcription factors. Covalent BTKi effectively impede this signaling cascade. Nevertheless, amino acid substitutions in BTK, especially at Cys-481, can confer resistance to covalent BTKi in B cell malignancies. Furthermore, CLL, MCL, and LPL exhibit diverse mechanisms of resistance, including 8p loss, mutations in *PLCG2*, *TRAF2*, *TRAF3*, *CCND1*, and *CARD11*. Image created with BioRender.com (accessed on 22 January 2024).

Baseline characteristics, such as del(17p), TP53 mutation, and complex karyotype, carry a higher risk of progression in ibrutinib-treated CLL [13,84]. Moreover, the acquired mechanisms of resistance were determined during treatment with BTKi or at relapse [13,85]. In this respect, acquired mutations of the *BTK* and phospholipase C gamma 2 (*PLCG2*) genes represent the most frequently reported resistance mechanisms in patients receiving covalent BTKi-based therapy (Figure 1). Mutations occur frequently at Cys-481, resulting in the replacement of cysteine by other amino acids (e.g., C481S, and C481R) [13]. Mutations at this site lead to the abrogation of covalent binding of ibrutinib, acalabrutinib, and zanubrutinib, with only transient inhibition of the mutant protein. Additionally, less frequent aminoacidic substitutions of PLCγ2 generally lead to gain-of-function of downstream signaling and promote BCR signaling despite BTK inhibition [85]. In a multicenter international retrospective study, the enrichment of 8p loss has been reported in BTKi-refractory patients in the absence of mutations of the *BTK* or *PLCG2* genes (Figure 1) [86]. This genetic aberration leads to haploinsufficiency of the TRAIL receptor (TRAIL-R), which causes leukemic cells to become resistant to TRAIL-induced apoptosis, independent of the mutational status of *BTK* and/or *PLCG2*. Moreover, *BIRC3* and *NFKBIE* mutations have been detected exclusively in ibrutinib relapsing patients carrying a wild-type *BTK* gene, suggesting that the aberrant activation of the canonical/noncanonical NF-κB pathway might be a possible mechanism of drug evasion [86]. A longer follow-up is needed to determine whether the presence of these mutations is associated with subsequent resistance to treatment with covalent BTKi. Other genetic alterations may complement *BTK* mutations in inducing BTKi resistance. For example, the transcription factor EGR2 was found to be almost exclusively mutated in relapsed patients carrying *BTK* mutations [86]. EGR2 is activated by ERK phosphorylation upon BCR stimulation, suggesting that EGR2 mutations may lead to constitutively dysregulated BCR signaling that, together with the existing *BTK*/*PLCG2* mutations, results in resistance to covalent BTKi (Figure 1).

Mutations of *BTK* and *PLCG2* play a role in acquired resistance to covalent BTKi also in MCL [87]. On the other hand, primary resistance to covalent BTKi in MCL may be due to elevated cyclin D1 levels, which can also arise from genomic deletions or point mutations in the 3’-untranslated region, producing shorter and more stable cyclin D1 transcripts (Figure 1) [88]. In addition, specific mutations (E36K, Y44D, or C47S) in *CCND1* increase cyclin D1 protein levels, causing defective proteolysis and promoting resistance to ibrutinib in MCL cell lines (Figure 1) [89]. Both mutant *CCND1* and overexpression of the wild-type gene have been shown to confer increased resistance to ibrutinib. Notably, the Y44D mutation of *CCND1* is associated with resistance even at high ibrutinib concentrations (5–10 μmol/L).

Moreover, MCL cell lines responsive to ibrutinib exhibit continuous BCR signaling, which leads to the activation of the classical NF-κB pathway via BTK [90]. In contrast, a possible mechanism of resistance to ibrutinib in MCL is represented by the hyper-activation of the alternative NF-κB pathway, which is independent of BTK signaling [90,91]. Somatic mutations identified in resistant MCL cell lines, including nonsense mutations in tumor necrosis factor receptor-associated factor 2 (*TRAF2*) and deletions in *TRAF3*, indicate a shift to the alternative NF-κB pathway (Figure 1) [90]. These mutations lead to loss-of-function in negative regulators of the alternative NF-κB pathway, promoting stabilization of the MAP3K14 enzyme. This stabilization facilitates the processing of p100 to nuclear factor kappa B subunit 2 (NFKB2), resulting in continuous activation of the alternative NF-κB pathway. The genomic profiling of archived MCL tumor samples has identified recurrent mutations in the *TRAF2*, *BIRC3*, and *MAP3K14* genes, suggesting a dependence on either the BCR-BTK-NF-κB (classical) or MAP3K14-NF-κB (alternative) pathways for MCL pathogenesis [90].

Recently, whole-exome sequencing of MCL samples has revealed recurrent mutations in various genes, including those already known to be mutated in MCL, such as *ATM*, *MEF2B*, and *KMT2D* [92]. Furthermore, this approach has identified novel mutated genes, including *CARD11*, encoding a scaffold protein required for BCR-induced NF-κB activation [92,93]. *CARD11* mutations were found in 5-15% of additional MCL cases and, when overexpressed in vitro, conferred resistance to ibrutinib and lenalidomide, indicating continuous activation of the NF-κB pathway, irrespective of BTKi (Figure 1) [92]. These findings offer new insights into ibrutinib resistance mechanisms in MCL.

Acquired resistance mechanisms to covalent BTKi in LPL include 8p loss, as well as aminoacidic substitutions of Cys-481 of *BTK* and *PLCG2* mutations [94]. Furthermore, *CXCR4* is the second most frequently mutated gene in LPL, with mutations occurring in up to 40% of patients [95]. In a physiological context, the interaction between the surface receptor CXCR4 and the chemokine CXCL12 leads to the activation of Akt signaling, initiating the transduction of anti-apoptotic stimuli [96]. Truncating mutations of the C terminal domain of CXCR4 cause defective internalization of the receptor upon binding CXCL12, resulting in a gain-of-function of CXCR4 [97]. Consistently, gain-of-function mutations of CXCR4 lead to the constitutive activation of Akt signaling, resulting in sustained survival signals for cancer cells [98]. Based on the mutation profile of *MYD88* and *CXCR4*, LPL patients may be divided into four subgroups, to predict their response to covalent BTKi [73,99]: (i) *MYD88*^mut^*CXCR4*^wt^, which is the most prevalent subtype and is characterized by LPL bearing the *MYD88* mutation without *CXCR4* mutation. These patients typically exhibit favorable responses to covalent BTK inhibition, with a major response rate (MRR) of more than 90% [95]; (ii) *MYD88*^mut^*CXCR4*^mut^, which identifies LPL harboring both mutations, which are more prone to presenting with hyperviscosity and bone marrow involvement. In comparison to *CXCR4* wild-type tumors, *MYD88*^mut^*CXCR4*^mut^ display an extended time to major response, reduced PFS, and reduced response to covalent BTKi. Response to BTKi in this subgroup also depends on the type of *CXCR4* mutation: frameshift mutations lead to an MRR of ~80%, while nonsense mutations display a worse predictive value, with an MRR of 55% [95]; (iii) *MYD88*^wt^*CXCR4*^wt^, which, in contrast to tumors with *MYD88* mutations, identifies LPL with wild-type *MYD88* and *CXCR4*, follows a more aggressive course and is associated with decreased OS. Additionally, *MYD88*^wt^*CXCR4*^wt^ cases exhibit poor responsiveness to covalent BTKi (MRR to ibrutinib: near to 0%) [95]; (iv) *MYD88*^wt^*CXCR4*^mut^, a rare combination, which likely has the same prognosis as *MYD88*^wt^*CXCR4*^wt^ cases.

DLBCL primary resistance to ibrutinib is associated with the presence of activating mutations in CARD11 and inactivating mutations in TNFAIP3 (also known as A20), a negative regulator of NF-κB. These genes function downstream of BTK and thus enhance NF-κB activity independent of upstream BTK activity [39,40,100,101]. The *KLHL14* gene frequently undergoes inactivating mutations in mature B cell malignancies, particularly in the *MYD88* L265P, *CD79B* mutant (MCD) subtype of DLBCL, which heavily relies on BCR signaling for survival [102]. Despite its unclear pathogenic role and molecular function in DLBCL, recent evidence reveals that KLHL14 facilitates the turnover of immature glycoforms of BCR subunits, leading to reduced total cellular BCR levels. Loss of KLHL14 confers resistance to ibrutinib and enhances the assembly of the MYD88-TLR9-BCR (My-T-BCR) supercomplex, thereby promoting prosurvival NF-κB activation.

Investigations of secondary resistance to ibrutinib in DLBCL primarily rely on cell line models due to the lack of comprehensive long-term follow-up data. Interestingly, mutations in *BTK* and *PLCG2* were not identified; however, resistant cases showed upregulation of the PI3K/AKT/mTOR signaling pathway, leading to increased tumor cell survival [103]. Additionally, recent studies have revealed the role of epigenetic mechanisms in ibrutinib resistance, particularly in ABC DLBCL cell lines [104]. In this respect, RAC2, a small GTPase, is upregulated through the increased accessibility of its enhancer domain. RAC2 activates PLCγ2 independent of BTK, thereby resulting in the activation of NF-κB.

## 4. BTK Targeting Approaches to Overcome Covalent BTKi Resistance

Reversible and non-covalent BTKi have been developed and proven effective in both C481-mutated and unmutated BTK in preclinical and clinical studies (Table 1). Examples include vecabrutinib, fenebrutinib, nemtabrutinib (ARQ 531), and pirtobrutinib (LOXO-305) [105,106,107]. Pirtobrutinib, a highly selective and noncovalent reversible BTKi, received approval in January 2023 for the treatment of adult patients with R/R MCL in the US [108]. Pirtobrutinib has demonstrated enduring efficacy and a positive safety profile in heavily pretreated patients with R/R MCL, especially those who had previously received covalent BTKi therapy (Table 1) [109]. Remarkably, the median ORR in patients who experienced progressive disease on a prior covalent BTKi was 43%, leading to the approval via the Accelerated Approval Program by FDA. Additionally, patients with high-risk disease features, such as blastoid/pleomorphic variants, elevated Ki-67 index, and *TP53* mutations, also exhibited favorable responses to pirtobrutinib [109]. In addition, the BRUIN study demonstrated that pirtobrutinib is able to achieve an ORR of 62% in R/R CLL cases after multiple lines of treatment, with the majority of cases previously being treated with a covalent BTKi [110]. A recent update of the BRUIN study reported a high efficacy profile of pirtobrutinib in LPL, with a remarkable MRR of 64% in patients who previously received at least one line of therapy with covalent BTKi [111].

Remarkably, recent reports have identified various mutations causing acquired resistance to both noncovalent and certain covalent BTK inhibitors in CLL [13]. These mutations, such as V416L, A428D, M437R, T474I, and L528W in the tyrosine kinase domain of BTK, have been shown to impair binding to both non-covalent and covalent BTKi in vitro [112]. Interestingly, enrichment of the BTK-L528W mutation was observed in CLL patients receiving zanubrutinib compared to ibrutinib in observational studies, suggesting the potential for cross-resistance with reversible BTK inhibitors [113]. Despite this evidence, a recent update of the results of the BRUIN study reported that although the L528W and T474x BTK mutations occur more frequently in patients who underwent treatment with covalent BTKi, response to pirtobrutinib is high even in those who display such mutations [114].

Targeting BTK with proteolysis-targeting chimeras (PROTACs) represents a promising strategy to overcome resistance to BTKi in B cell neoplasms (Table 2) [13,115]. PROTACs represent a novel category of small molecules that employ two covalently linked ligands [115]. These ligands work in tandem to recruit both the target protein and E3 ubiquitin ligase, initiating and facilitating the proteasomal degradation of the target protein [116]. Several preclinical studies have demonstrated the effectiveness of PROTACs against in vitro mutant BTK-C481 cells, inducing BTK degradation through ubiquitin-mediated protein breakdown. Notably, NX-2127, the pioneering targeted protein degrader of BTK, exhibited its efficacy in preclinical studies by promoting the degradation of both wild-type and mutant BTK [117]. In addition to BTK degradation, NX-2127 also demonstrated a preclinical efficacy comparable to that of immunomodulatory drugs (IMiDs), such as lenalidomide and pomalidomide [118,119]. NX-2127 achieves this effect by catalyzing the ubiquitination of Ikaros (IKZF1) and Aiolos (IKZF3), thus leading to heightened T-cell activation [118,120,121]. While further research is required, the increased T-cell activity facilitated by NX-2127 has potential for addressing the immune dysfunction commonly associated with CLL [122]. Recently reported clinical results from a first-in-human phase I trial on NX-2127 involved 23 R/R CLL patients, with a median of six prior therapies (2–11), all of whom had previously undergone treatment with a covalent BTKi and/or BCL2 inhibitor venetoclax [117]. In this challenging patient cohort with limited therapeutic options, NX-2127 demonstrated an overall response rate (ORR) of 33% in 12 evaluable patients, with a median follow-up of 5.6 months [117]. These findings underscore the potential utility of BTK degraders, such as NX-2127, in patients refractory to multiple lines of therapy, irrespective of their BTK mutation status. Phase I clinical trials of BTK degraders in the treatment of B cell malignancies have been initiated and are currently ongoing (Table 2).

## 5. Conclusions and Perspectives

Great progress has been made in the treatment of B cell neoplasms, particularly in targeting BTK in CLL, MCL, and LPL. The first drugs developed to inhibit BTK activity were covalent BTKi, namely first-generation (ibrutinib) and second-generation (acalabrutinib, zanubrutinib) BTKi. The mechanism of action of covalent BTKi involves inhibiting the kinase activity of the molecule via the binding of the drugs to the amino acid residue Cys-481, located in the ATP-binding pocket. Despite the significant efficacy demonstrated in CLL, MCL, and LPL, primary and secondary resistance phenomena have emerged in patients treated with covalent BTKi.

Third-generation inhibitors, such as pirtobrutinib, can overcome the resistance to covalent inhibitors developed by B cell neoplasms because they have a non-covalent mode of action, featuring a reversible binding to BTK. Pirtobrutinib has indeed shown excellent results in terms of efficacy in CLL, MCL, and LPL, even in diseases refractory to treatment with second-generation inhibitors. Furthermore, as a perspective for the future, BTK degraders have demonstrated their effectiveness in CLL, and several studies are underway to test these drugs in other B cell neoplasms.

## Figures and Tables

**Table 1:** Ongoing clinical trials with non-covalent BTKi in B cell malignancies (from www.clinicaltrial.gov, accessed on 15 January 2023).

NCT Identifier	Phase	Condition	Intervention
NCT05734495	II	LPL	Pirtobrutinib + venetoclax
NCT05317936	II	CLL	Pirtobrutinib + venetoclax
NCT05254743	III	CLL	Pirtobrutinib vs. ibrutinib
NCT05529069	II	R/R MCL	Pirtobrutinib + venetoclax
NCT05006716	I/II	R/R B cell malignancies, namely CLL, MM, MCL, LPL, MZL	Pirtobrutinib + LOXO-338
NCT04965493	III	R/R CLL	Pirtobrutinib + venetoclax + rituximab
NCT03740529	I/II	R/R B cell malignancies, namely CLL, MCL, LPL, MZL	Pirtobrutinib ± venetoclax ± rituximab
NCT05990465	I	R/R NHL, namely MCL, DLBCL, MZL, BL, FL	Pirtobrutinib + LV20.19 CAR T cells
NCT05833763	II	BTKi-refractory MCL	Pirtobrutinib + glofitamab + obinutuzumab
NCT04849416	II	R/R B cell malignancies, namely CLL, MCL, DLBCL, MZL	Pirtobrutinib
NCT04662255	III	R/R BTKi naïve MCL	Pirtobrutinib vs. ibrutinib/acalabrutinib/zanubrutinib
NCT05536349	II	Treatment-naïve CLL/RS	Pirtobrutinib + venetoclax+ obinutuzumab
NCT05677919	II	Treatment-naïve CLL	Pirtobrutinib + venetoclax
NCT04666038	III	R/R CLL	Pirtobrutinib vs. BR/idelalisib + rituximab
NCT03162536	I/II	R/R B cell malignancies, namely CLL, MCL, LPL, DLBCL, MZL, FL, RS	Nemtabrutinib
NCT05683717	I	R/R B cell malingnancies, including CLL, DLBCL, other NHL	TT-01488
NCT05275504	I	R/R B cell malignancies, including CLL, LPL, FL, MZL, DLBCL, other NHL	TT-01488
NCT05023980	III	Treatment-naïve CLL	Pirtobrutinib vs. BR

Abbreviations: LPL, lymphoplasmacytic lymphoma; CLL, chronic lymphocytic leukemia; R/R, relapsed/refractory; MCL, mantle cell lymphoma; MM, multiple myeloma; MZL, marginal zone lymphoma; NHL, non-Hodgkin lymphoma; DLBCL, diffuse large B cell lymphoma; BL, Burkitt lymphoma; FL, follicular lymphoma; RS, Richter syndrome; BTKi, Bruton tyrosine kinase inhibitor; CAR, chimeric antigen receptor.

**Table 2:** Ongoing clinical trials with BTK degraders in B cell malignancies (from www.clinicaltrial.gov, accessed on 15 January 2023).

NCT Identifier	Phase	Condition	Intervention
NCT04830137	I	CLL, MCL, LPL, MZL, FL, DLBCL, PCNSL	NX-2127
NCT05294731	I	CLL, MCL, LPL, MZL, FL, DLBCL, RS	BGB-16673
NCT05131022	I	CLL, MCL, LPL, MZL, FL, DLBCL, PCNSL	NX-5948
NCT05753501	I	CLL, MCL, LPL, MZL, FL, DLBCL	ABBV-101
NCT05006716	I/II	CLL, MCL, LPL, MZL, FL, DLBCL	BGB-16673
NCT05780034	I	R/R NHL, namely CLL, MCL, LPL, MZL, FL, DLBCL	AC676

Abbreviations: LPL, lymphoplasmacytic lymphoma; CLL, chronic lymphocytic leukemia; R/R, relapsed/refractory; MCL, mantle cell lymphoma; MZL, marginal zone lymphoma; NHL, non-Hodgkin lymphoma; DLBCL, diffuse large B cell lymphoma; FL, follicular lymphoma; RS, Richter syndrome; PCNSL, primary central nervous system lymphoma.
